# An analysis of the changes in soluble hydrogenase and global gene expression in *Cupriavidus necator* (*Ralstonia eutropha*) H16 grown in heterotrophic diauxic batch culture

**DOI:** 10.1186/s12934-015-0226-4

**Published:** 2015-03-25

**Authors:** Bat-Erdene Jugder, Zhiliang Chen, Darren Tan Tek Ping, Helene Lebhar, Jeffrey Welch, Christopher P Marquis

**Affiliations:** School of Biotechnology and Biomolecular Sciences, University of New South Wales, Sydney, 2052 Australia; Systems Biology Initiative, University of New South Wales, Sydney, 2052 Australia

**Keywords:** Soluble hydrogenase, *Ralstonia eutropha*, *Cupriavidus necator*, Heterotrophic batch culture, Controlled bioreactor, Transcriptome

## Abstract

**Background:**

Soluble hydrogenases (SH) are enzymes that catalyse the oxidation of molecular hydrogen. The SH enzyme from *Cupriavidus necator* H16 is relatively oxygen tolerant and makes an attractive target for potential application in biochemical hydrogen fuel cells. Expression of the enzyme can be mediated by derepression of the *hox* promoter system under heterotrophic conditions. However, the overall impact of *hox* derepression, from a transcriptomic perspective, has never been previously reported.

**Results:**

Derepression of hydrogenase gene expression upon fructose depletion was confirmed in replicate experiments. Using qRT-PCR, *hoxF* was 4.6-fold up-regulated, *hypF2* was up-regulated in the cells grown 2.2-fold and the regulatory gene *hoxA* was up-regulated by a mean factor of 4.5. A full transcriptomic evaluation revealed a substantial shift in the global pattern of gene expression. In addition to up-regulation of genes associated with hydrogenase expression, significant changes were observed in genes associated with energy transduction, amino acid metabolism, transcription and translation (and regulation thereof), genes associated with cell stress, lipid and cell wall biogenesis and other functions, including cell motility.

**Conclusions:**

We report the first full transcriptome analysis of *C. necator* H16 grown heterotrophically on fructose and glycerol in diauxic batch culture, which permits expression of soluble hydrogenase under heterotrophic conditions. The data presented deepens our understanding of the changes in global gene expression patterns that occur during the switch to growth on glycerol and suggests that energy deficit is a key driver for induction of hydrogenase expression in this organism.

**Electronic supplementary material:**

The online version of this article (doi:10.1186/s12934-015-0226-4) contains supplementary material, which is available to authorized users.

## Background

Hydrogenases are metalloenzymes that reversibly catalyse the oxidation or production of molecular hydrogen (H_2_) and are generally classified by the structure of their catalytic site [1]. Since they were first described in 1931 by Stephenson and Stickland [2], hydrogenases have been studied extensively with a view to their potential application in hydrogen-based energy systems. There have been reports of recent achievements in designing electrochemical fuel cells that exploit the H_2_ oxidation activity of [Ni-Fe]-hydrogenases [3-5]. Application of these enzymes in electrochemical fuel cells offers potential advantages over traditional platinum catalysts. However, these enzymes may be sensitive to environmental parameters such as oxygen, carbon monoxide, pH and temperature in the fuel cell environment.

Amongst a number of promising candidates for application in the oxidation of H_2_ is a soluble [Ni-Fe] uptake hydrogenase (SH) produced by *Cupriavidus necator* (more commonly known by its previous name, *Ralstonia eutropha*). This enzyme has been proven to demonstrate tolerance to O_2_ and CO [6,7]. Two growth conditions have been reported to lead to induction of expression of hydrogenases in *C. necator* cells: (1) autotrophic and (2) heterotrophic growth [8]. Induction under autotrophic conditions requires the presence of H_2_ mixed with other gases, mostly CO_2_ and O_2_; this technique has been employed widely to achieve expression of the hydrogenases (membrane-bound hydrogenase (MBH) and SH) found in *C. necator*. Induction under heterotrophic conditions has been conveniently achieved by using a defined Fructose Glycerol Nitrogen (FGN) medium whereby substrate shift occurs from the preferentially utilised carbon source (fructose), to the less-preferred glycerol resulting in derepression of the *hox* regulon, which contains the hydrogenase structural and auxiliary genes [9,10]. This feature allows for a substantially safer fermentation process to isolate biomass with active soluble hydrogenases, avoiding an autotrophic fermentation process which requires the use of hazardous gas mixtures of H_2_, O_2_ and CO_2_. More importantly, it has been previously found that MBH and SH with a higher specific activity were produced in cells growing using the heterotrophic process than enzymes produced in autotrophic conditions [11,12].

The SH resides in the cytoplasm and belongs to the family of bidirectional heteromultimeric cytoplasmic [Ni-Fe]-hydrogenases. The SH was thought to be a heterotetrameric enzyme until the recent discovery of two additional HoxI_2_ subunits [13]. It has two functionally distinct moieties: the hydrogenase moiety comprised of HoxH and HoxY; and the NADH dehydrogenase (diaphorase) moiety consisting of HoxF and HoxU. The large subunit of the hydrogenase moiety (HoxH) harbours the [Ni-Fe] active site where H_2_ is oxidised. The electrons released are then transferred via the Fe-S clusters in HoxY and HoxU to FMN-b, which is located in the large subunit (HoxF) of the diaphorase moiety. HoxF also contains a Fe-S cluster and is responsible for the reduction of NAD^+^ to NADH, which is used mainly for autotrophic CO_2_ fixation [6]. HoxI_2_ subunits were proposed to be responsible for providing a NADPH binding site [13]. The SH operon is reportedly long (~10 kb) and comprises the structural genes (*hoxFUYH*, nucleotides 79,712–84,331) of the heterotetrameric hydrogenase, two accessory genes (*hoxI*_*2*_, nucleotides 84,315–85,338), and a partial set of maturation *hyp* genes (*hypA2, hypB2, hypF2*, nucleotides 85,449–85,790) [14]. For the maturation of fully active SH, not only this set of *hyp* genes, but also *hypC1, hypD1*, and *hypE1* are required [6,15].

In the present study, two SH genes, *hoxF* and *hypF2*, as well as a regulatory gene, *hoxA*, were chosen as indicators for investigating the expression of the SH genes from *C. necator* under heterotrophic conditions in controlled batch fermentations. The *hoxF* gene is the first gene within the SH operon on the megaplasmid pHG1 and encodes for the NAD-reducing hydrogenase diaphorase moiety large subunit (Gene ID: 2656814), whereas *hypF2* is the last gene of the same operon (Gene ID: 2656550). The rationale for choosing the first and last genes of the SH operon was to examine whether the ORF’s are effectively expressed at the same level and to examine the transcript stability. The *hoxA* gene (Gene ID: 2656440) encodes the HoxA protein which acts as a transcriptional activator (NtrC family) of the hydrogenase genes and lies on a different transcript [16]. Finally, a full analysis of the derepression process was performed by undertaking a comparative whole transcriptome experiment, comparing global gene expression at two different stages of the diauxic batch fermentation process.

## Results and discussion

### Heterotrophic growth in batch fermentation

**Figure 1:**
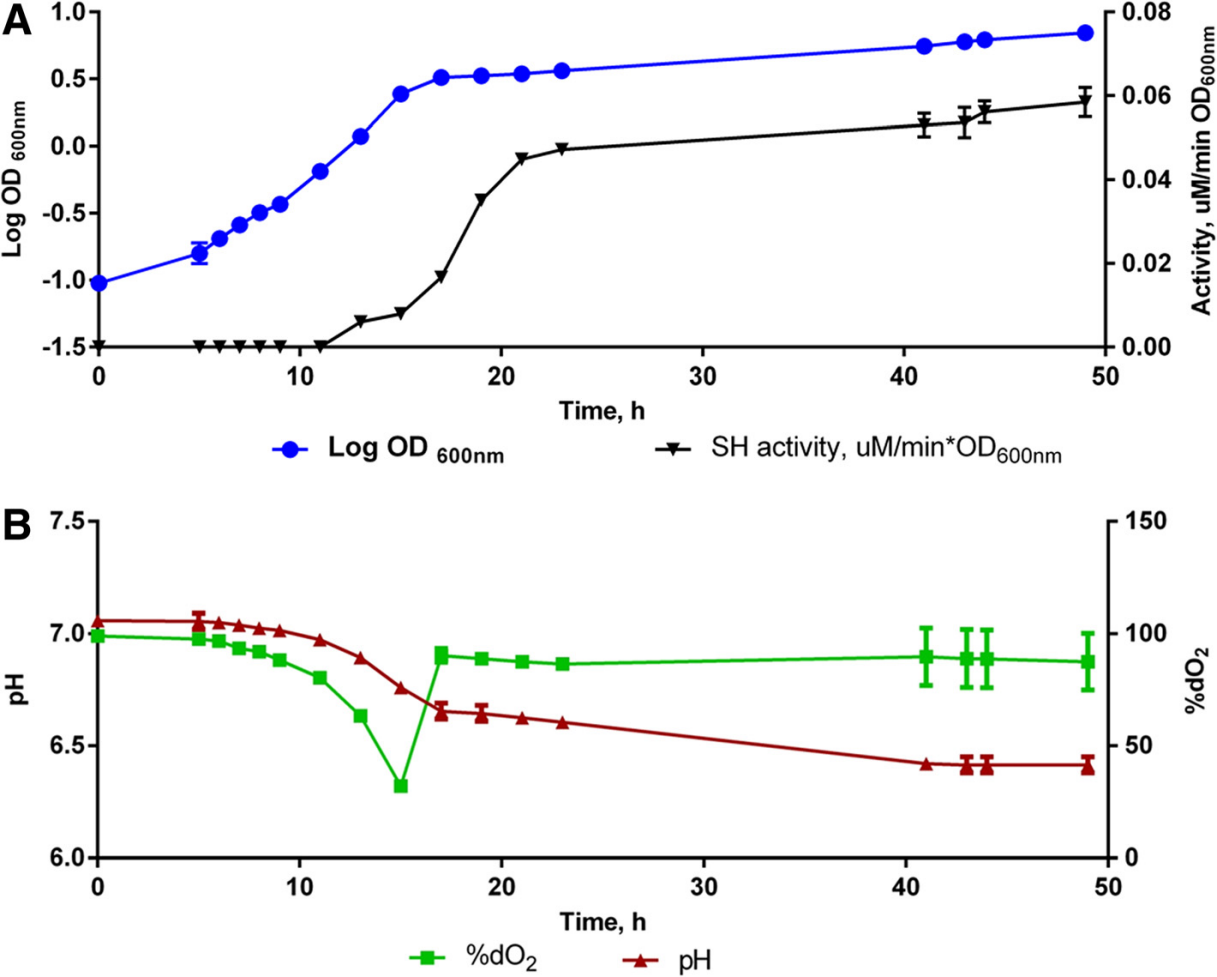
**Heterotrophic growth of** ***C. necator*** **H16 cells during a 48 h bioreactor fermentation. A)** Log_10_ OD_600nm_ and soluble hydrogenase activity (by NAD+ reduction) with time; **B)** dO_2_ and pH variation with time. These graphs are based on three biological replicates and represent their mean values with standard deviation given.

The aerobic growth kinetics of *C. necator* H16 was examined in a controlled 7 L bioreactor (operating volume 5 L). Cell growth commenced on fructose until approximately 15 h post-inoculation, whereupon growth switched to glycerol [10]. This shift can be identified by the sudden increase in dissolved oxygen (dO_2_) concentration and the slowing of cell growth (Figure 1). At a similar time point, the declining trend of pH slows (again a characteristic of slower carbon source metabolism). The pH does not fall below 6.4 as it was automatically regulated by alkali addition to the reactor, triggered at a setpoint of pH 6.4. The specific growth rates (μ) of the cells growing on fructose and glycerol were determined by linear regression of the pre and post-induction phase data, respectively, and found to be 0.31 h^−1^ and 0.18 h^−1^ respectively. The shift to slower growth on glycerol coincides with the increase in the SH activity level (Figure 1).

### Up-regulation of expression of *hoxF*, *hypF2* and *hoxA* genes in response to growth on glycerol (derepression of the soluble hydrogenase genes)

qRT-PCR was employed to analyse the levels of expression of the *hoxF*, *hypF2* and *hoxA* genes. All quantitative PCR results were normalised against the expression of the *gyrB* housekeeping gene, which was shown to be the most stable of the three reporter genes tested (*gyrB, 16S rRNA* and *rpoD1*)*.* The relative expression levels of the genes of interest that were assayed using qRT-PCR are presented in Table 1. The qRT-PCR experiments confirmed the observation of an anticipated significant increase in transcript level of the soluble hydrogenase genes in *C. necator* cells under derepressing growth conditions.

**Table 1:** **REST 2009 output for all genes assayed using qRT-PCR**

**Gene**	**Type**	**Expression**	**Std. Error**	**95% C.I.**	**P(H1)**	**Result**
*gyrB*	REF	1				
*hoxF*	TRG	4.6	3.663 - 5.539	2.945 - 6.720	0	UP
*hypF2*	TRG	2.5	1.407 - 3.677	0.938 - 5.930	0.035	UP
*hoxA*	TRG	4.4	3.814 - 4.959	3.527 - 5.467	0.002	UP

95% C.I. represents 95% confidence intervals for expression ratios. P(H1) is the p-value that indicates the significance of the observed regulation. Result represents the significance of up- or down-regulation of the target genes. *gyrB* was used as an endogenous reference gene. REF represents a reference gene, whereas TRG – a target gene.

The gene *hoxF* (Gene ID: 2656814) encodes the diaphorase moiety large subunit of the SH from *C. necator* H16 and is the first gene residing on the SH operon. The gene *hypF2* (Gene ID: 2656550) encodes HypF2, an auxiliary protein, which is essential for the synthesis of the two cyanide groups at the active site of [Ni-Fe] hydrogenases from carbamoyl phosphate [17]. The *hypF2* is located at the last ORF position within the SH operon. The main regulatory gene that controls both MBH and SH operons from *C. necator* is *hoxA* (Gene ID: 2656440) [18]. It has also been determined that MBH and SH transcripts are reduced significantly in *hoxA* mutants [19]. The product of *hoxA* is a response regulator-type transcriptional activator [20].

Statistically significant differences in transcript levels of the target genes in comparison to the reference gene were observed between the repressed and derepressed state. The gene *hoxF* was 4.6-fold up-regulated in the post-induction cells (S.E. range is 3.663 - 5.539, P(H1) = 0.000). The gene *hypF2* was also up-regulated in the cells grown on glycerol by a mean factor of 2.5 (S.E. range is 1.407 - 3.677). This increase in transcript level is also statistically significant when compared to that of the reference gene (P(H1) = 0.035). The regulatory gene *hoxA* was up-regulated by a mean factor of 4.4 (S.E. range is 3.814 - 4.959, P(H1) = 0.002).

The first investigation of the transcriptional regulation of the hydrogenase genes in *C. necator* was performed by Schwartz *et al.* using primer extension analysis [16]. According to their study, a significant increase in levels of SH and MBH mRNA’s were observed in cells growing on glycerol (on a FGN medium) upon derepression of hydrogenase expression, and the promoter activity of both MBH and SH were dependent on a sigma factor σ^54^ and HoxA. Relative transcript abundance of both SH and MBH of the heterotrophically grown cells were reported to be significantly lower than that of the autotrophically growing cells. Similar findings were reported in a more recent study by Kohlmann *et al.* 2011 [21] using *in vivo* metabolic labelling combined with the shotgun gel-based liquid chromatography-mass spectrometry (GeLC-MS) analysis where the expression profile of both the MBH and SH genes in lithoautotrophically grown cells was observed to be strongly up-regulated (approximately 13 and 20 fold, respectively). The observed changes in the present study with regards to the expression of SH genes in the heterotrophically grown cells using a qRT-PCR method are in accordance with the kinetics of transcript abundance of SH genes found from their promoter activity measurements.

### Global transcriptional profiling of *C. necator H16* under hydrogenase-derepressing conditions

Genome-wide transcriptional analysis was performed to investigate genes that are differentially expressed in response to derepression of hydrogenase genes expression. For this purpose, we analysed transcripts of two different growth phases of the heterotrophically grown cells: the repressed and derepressed states where fructose and glycerol is utilised, respectively. Across all eight samples, including two technical replicates of two growth phases from two biological replicates, between 23,199,739 and 33,787,998 pairs of reads were generated by the Illumina HiSeq2000 sequencer. Overall 98.9% to 99.15% alignment rates were obtained by TopHat (version 2.0.4) when mapped to the reference genome of *C. necator* H16 (NCBI accession number NC_008313.1, NC_008314.1 and NC_005241.1) after quality filtering and trimming. The Python script htseq-count was then used to generate the read counts using BAM files output by TopHat. The count files were then merged into a count table containing read-count information for all samples.

The two R packages, edgeR and DESeq2, were applied to the count table separately to calculate the gene expression levels under the two different growth conditions. edgeR reported a total of 2744 up-regulated SDE genes and 2211 down-regulated SDE genes in 34 hours, while DESEq2 reported 2609 up-regulated SDE genes and 2459 down-regulated SDE genes.

**Figure 2:**
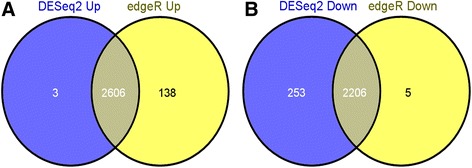
**Venn Diagram of Differentially Expressed Genes. (A)** - 2606 genes up-regulated at 34 hours identified in edgeR and DESeq2 analyses. **(B)** - 2206 genes down-regulated at 34 hours identified in edgeR and DESeq2 analyses.

**Figure 3:**
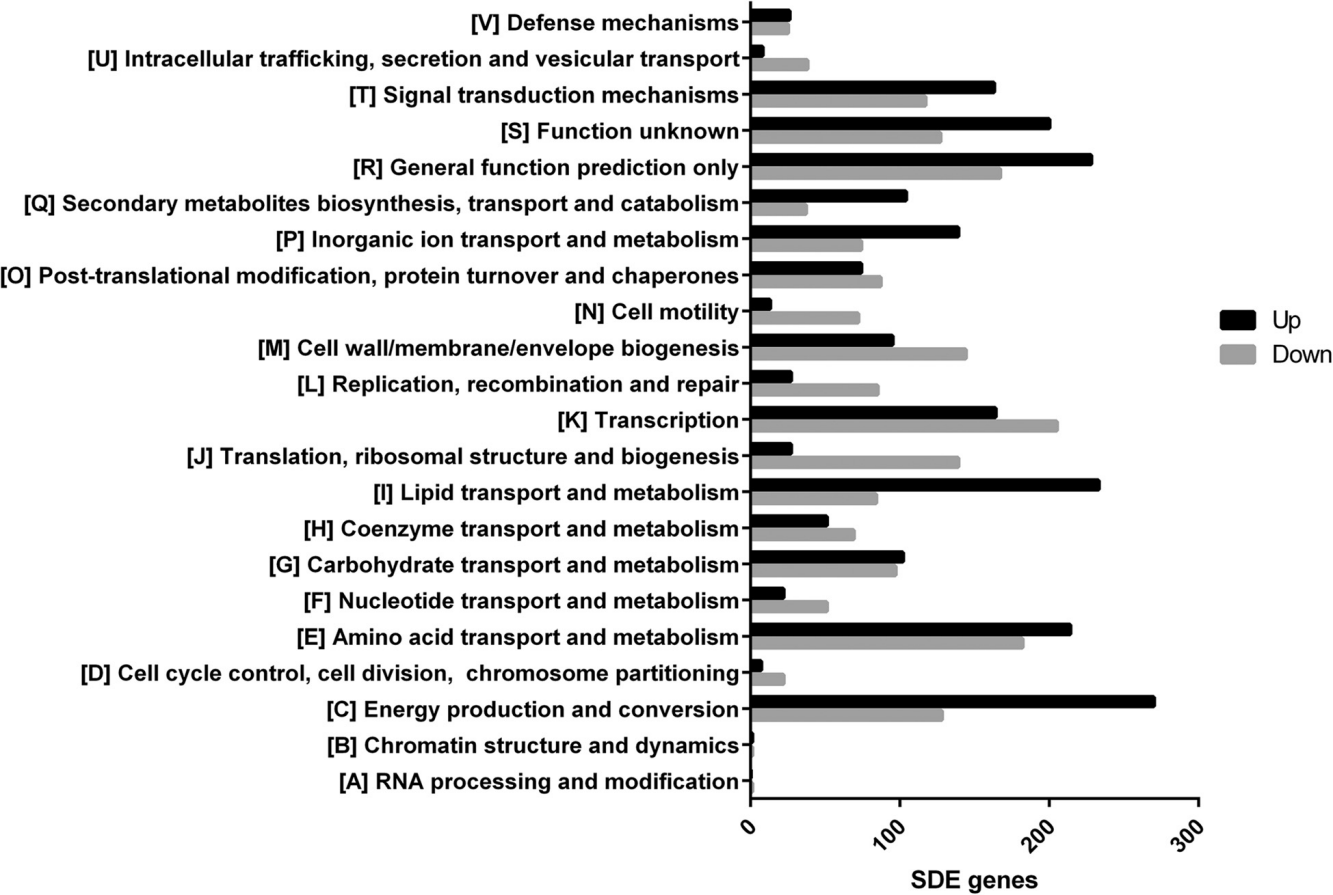
**Clusters of Orthologous Groups (COGs) of the up and down regulated genes during derepression of hydrogenase genes.** The numbers of up-regulated SDE genes belonged to each COG category are represented in black bars, whereas down-regulated genes are in grey bars.

The lists of 2606 up-regulated and 2206 down-regulated SDE genes at 34 hours were reported by both edgeR and DESeq2 (Figure 2A, B). Of the overexpressed genes, 1641 genes were located on chromosome 1 (63%), 852 were on chromosome 2 (33%) and 113 were on the megaplasmid pHG1 (4%). This pattern of gene distribution by replicon is consistent with the findings of previous proteomic studies for monoauxic growth [21,22]. The COG analysis of the up and down regulated SDE genes classified these genes into 22 functional categories (Figure 3, Additional file 1: Table S1 and Additional file 2: Table S2). The top five COG functional categories containing most of the up-regulated SDE genes are: [C] Energy production and conversion (272 genes), [E] Amino acid transport and metabolism (224 genes), [I] Lipid transport and metabolism (227 genes), [R] General function prediction only (331 genes) and [S] Function unknown (198 genes).

In the down-regulated SDE gene list, the top five COG categories are: [K] Transcription (218 genes), [E] Amino acid transport and metabolism (187 genes), [R] General function prediction only (202 genes), [M] Cell wall/membrane/envelope biogenesis (143 genes) and [J] Translation, ribosomal structure and biogenesis (147 genes). Comprehensive gene expression profiling performed in this study revealed commonly observed phenomena of extensively inhibited macromolecule synthesis due to slower growth on glycerol during the second phase of diauxic growth. Such extensive reprogramming of gene expression was observed in the transcripts from the cells grown in glycerol. The most notably affected down-regulation was observed in the transcription and translation apparatus genes including various ribosomal proteins (for example genes in the RPL family), numerous transcriptional regulators (genes encoding NagC, ArsR, MarR, GntR, GlvR, TetR, LysR-family regulators, RNA polymerase factor sigma-32 and 70), translation factors (translation initiation factor IF-1 and 2, elongation factor Ts, Tu, G and P) and RNA polymerase subunits (*rpoABCZ* and *fliA*). Similar down-regulation of macromolecule biosynthesis for stringent responses was observed in *E. coli*, as growth transitioned in to the stationary phase [23].

**Figure 4:**
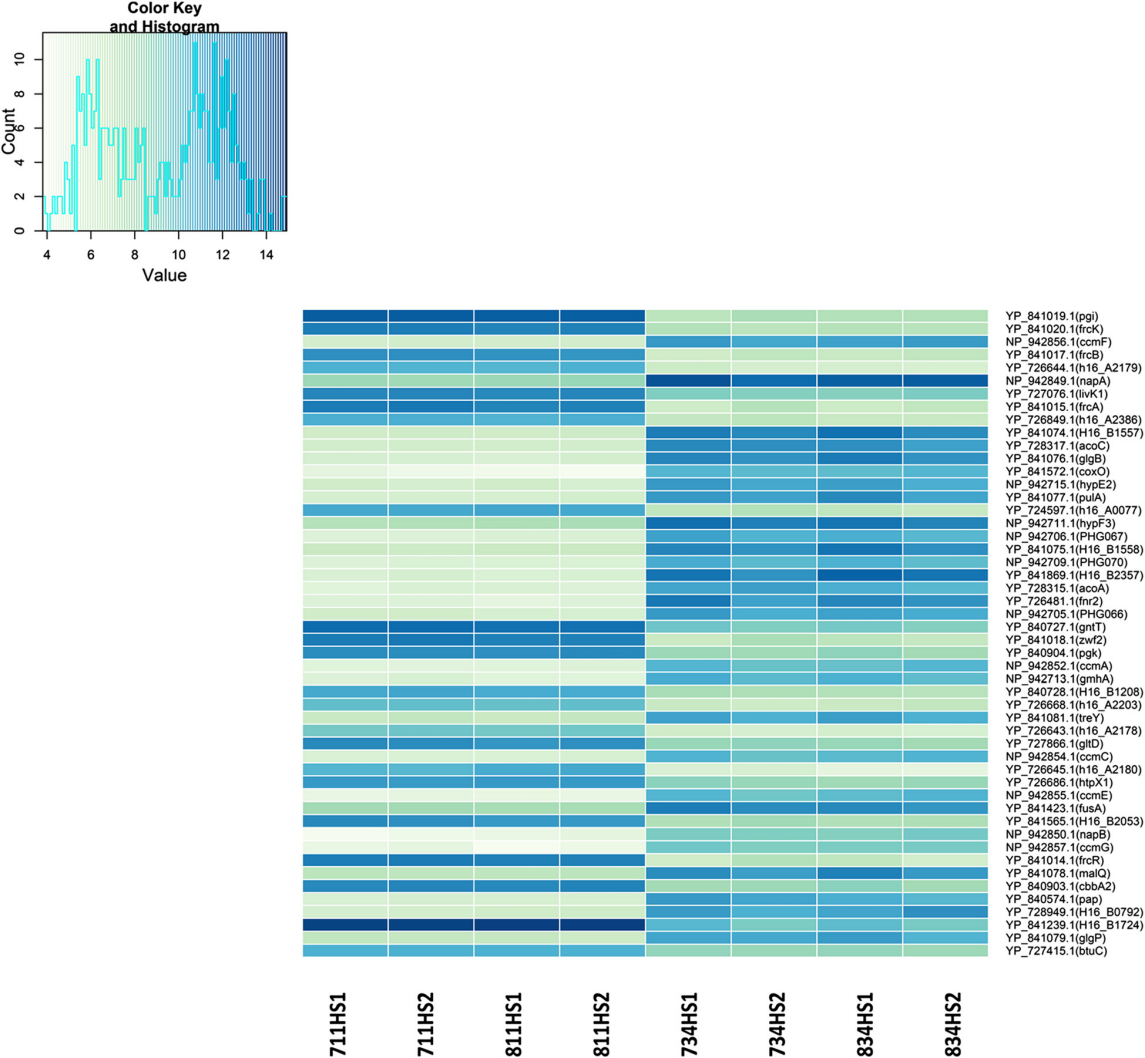
**Heatmap showing the expression data of the 50 most differently expressed genes.** The data is ordered by absolute values of fold-changes of raw counts calculated from variance stabilizing transformation. 711HS1 and 711HS2, as well as 811HS1 and 811HS2 are technical replicates of two biological replicates of pre-induction samples, whereas 734HS1, 734HS2, 834HS1 and 834HS2 are technical and biological replicates of post-induction samples.

Moreover, we identified the list of 50 most differentially expressed (DE) genes (Figure 4) by selecting the 50 highest absolute fold-change values and analysed these genes across all eight samples in both *hox* operon repressed and derepressed conditions. For clarity, the genes overexpressed in the later phase (growth on glycerol) are hereafter discussed as overexpressed/up-regulated, whereas those comparatively overexpressed in the fructose growth phase are herein referred to as down-regulated.

### Changes in global gene expression with a focus on hydrogenase biosynthesis

**Figure 5:**
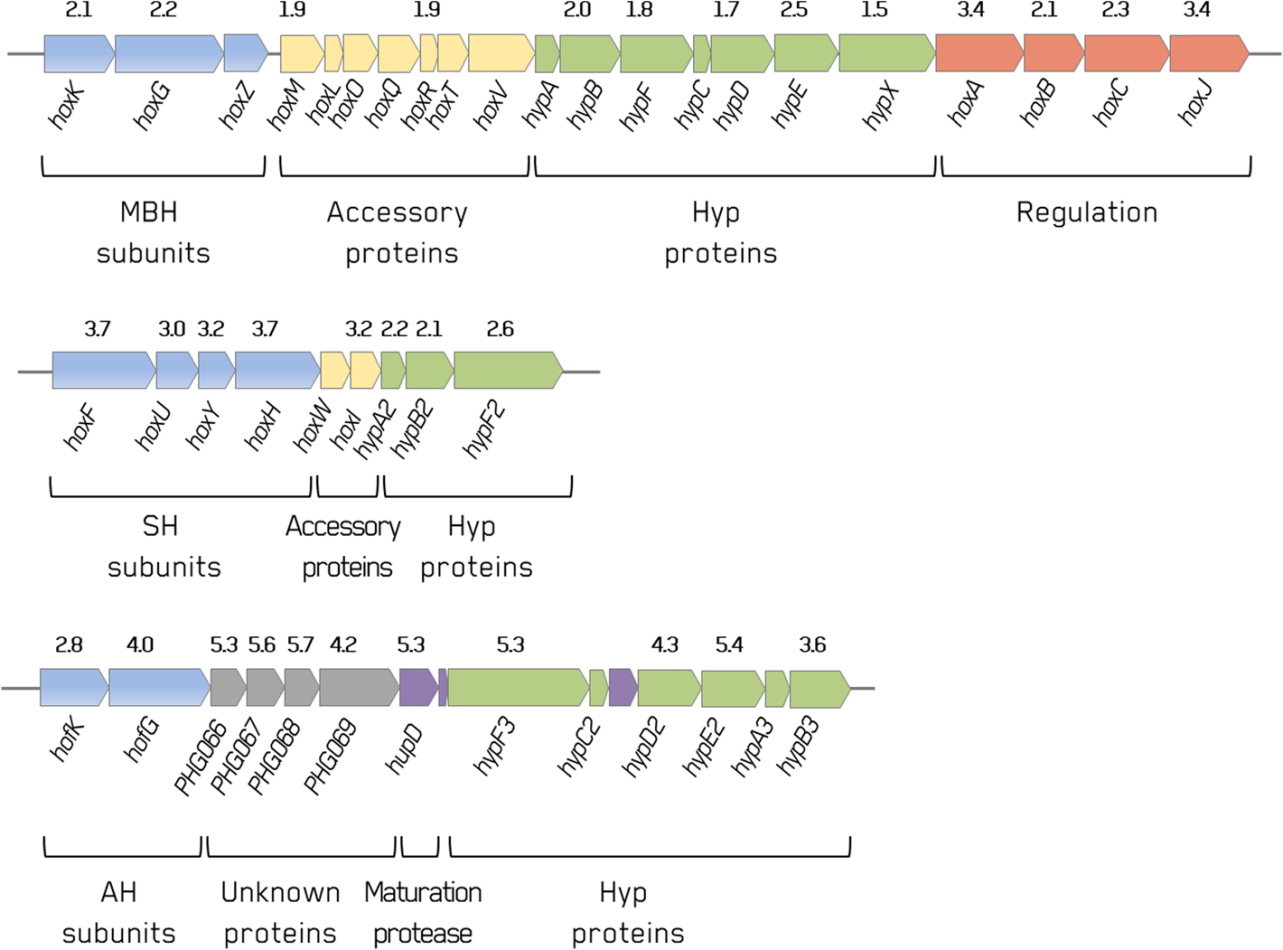
**Overview of the hydrogenase operons of** ***C. necator.*** The three operons, where four hydrogenases (MBH, SH, RH and AH) are located, are depicted with respective genes. Results of the transcriptome analyses are given in log2 fold changes on top of each gene detected.

In *C. necator* H16, the genes encoding the three uptake hydrogenases occupy a 90-kb region of the megaplasmid pHG1 [24]. Genes for MBH and SH reside on two major operons: a MBH operon (21-kb) and a SH operon (10-kb), respectively, separated by 59-kb. Several structural and accessory genes are involved in the complex expression of both MBH and SH: 21 genes and 13 genes on the MBH and SH operons, respectively. As expected most of the genes encoding the subunits of both MBH (*hoxKG*) and SH (*hoxFUYHI*) were positively regulated (Figure 5). However, *hoxZ* of the MBH was not found and also only two (*hoxM* and *hoxR*) of seven MBH-specific genes could be detected. Aside from the structural genes, most of the maturation genes required for the biosynthesis and maturation of the uptake hydrogenases were demonstrated to be overexpressed in the post-induction phase. The SH maturation *hyp* genes set (*hypA2, hypB2, hypF2*) was overexpressed, and the MBH maturation genes *hypB1, hypF1, hypD1, hypE1* were detected. The expression of both the MBH and the SH responds to cellular energy limitation and a signal transduction complex consists of HoxJ protein and the RH [25,26]. The genes encoding the subunits of RH were (*hoxCB*) identified with other key regulatory genes *hoxA* (a response regulator) and *hoxJ* (histidine protein kinase) that were also up-regulated in accordance with previous proteome studies [21,22]. Two strong *hox* promoters, P_MBH_ and P_SH_, were identified upstream of the *hoxK* and *hoxF* genes, respectively [16]. Up-regulation of the uptake hydrogenase system detected in the present study reflects induction of these two promoters and corresponding increases in the MBH and SH transcripts previously reported [16]. Both promoters are recognized by the alternative sigma factor σ^54^ (RpoN) of the RNA polymerase, and gene *h16_A0386* encoding Sigma54 (RpoN) modulation protein was also up-regulated.

One striking finding in our study was that the most highly expressed hydrogenase in the glycerol-grown cells was the actinobacterial hydrogenase (AH), a recently discovered novel Group 5 hydrogenase in *C. necator* H16 (Figure 5) [27]. The AH is an oxygen tolerant, cytoplasmic [Ni-Fe]-uptake hydrogenase encoded by two subunit genes (*hofKG*) clustered with a set of hydrogenase maturation genes (*hypA3B3C2D2E2F3* and *hupD*), and four open reading frames coding for proteins of unknown function (*PHG066, 67, 68, 69*). In the present study, the maturation genes *hypE2* and *F3*, also *PHG066* and *PHG067* genes as well as *PHG070* (putative hydrogenase-specific C-terminal protease) were highly expressed and listed in the top 50 genes (Figure 4). The two subunits genes, *hofK* (*PHG064*, [NiFe] hydrogenase small subunit) and *hofG* (*PHG065*, [NiFe] hydrogenase large subunit) were also up-regulated 2.86 and 4.02-fold, respectively (Additional file 1: Table S1). The strong induction of the AH gene expression in the *C. necator* H16 cells growing in the heterotrophic media is consistent with previous reports showing that it is more inducible under carbon-limited aerobic growth compared to growth under lithoautotrophic conditions [27].

### Central carbohydrate metabolic changes upon carbon/energy source shift

Among the top 50 DE genes are seven overexpressed genes that are involved in the starch and sucrose metabolism pathway (KEGG pathway reh 00500) in *C. necator* H16 (Figure 4). These are *H16_B1557* (glycosidase-like protein), *glgB* (glycogen branching enzyme), *pulA* (Type II secretory pathway, pullulanase PulA), *H16_B1558* (trehalose synthase), *treY* (maltooligosyl trehalose synthase), *malQ* (4-Alpha-glucanotransferase) and *glgP* (glucan phosphorylase). The roles of this set of genes are likely to be associated with carbohydrate storage and stress response.

The observed up-regulation of genes *h16_A1789* (glycerol trinitrate reductase, 1.45-fold), *h16_A2507* (glycerol kinase, 2.04-fold) and *h16_A2508* (glycerol-3-phosphate dehydrogenase, 2.28-fold) can be attributable to glycerol metabolism by the cells after the carbon source shift (Additional file 1: Table S1).

As may be expected, the genes responsible for fructose transport and metabolism were highly expressed in the cells under repressing condition (growth on fructose). The up-regulation of *frcA* (ABC transporter ATPase), *frcB* (ABC transporter periplasmic protein) and *frcK* (fructokinase), *frcR* (NagC-family transcriptional regulator) genes can be attributable to the growth state where fructose is the preferential carbon source for the cells (Figure 4). Additionally, *cbbA2* (fructose-1,6-bisphosphate aldolase), *gntT* (gluconate transporter), *zwf2* (glucose-6-phosphate 1-dehydrogenase), *pgi* (glucose-6-phosphate isomerase) and *pgk* (phosphoglycerate kinase) genes categorised in the functional group of carbohydrate transport and metabolism were up-regulated in the cells grown with fructose.

In the list of the top 50 DE genes are also two overexpressed genes in the second phase of growth, *acoA* (acetoin dehydrogenase E1 component alpha-subunit) and *acoC* (branched-chain alpha-keto acid dehydrogenase subunit E2), which are involved in catabolism of acetoin, a product of the fermentative metabolism of many prokaryotes. The acetoin dehydrogenase E1 component beta-subunit-encoding *acoB* gene was also similarly up-regulated by 5.57-fold. The induction of *aco* genes during the growth of *C. necator* on acetoin has been previously reported [28] and this is associated with pyruvate metabolism in the bacteria (KEGG pathway reh 00620). To avoid overacidification of the growth condition, pyruvate is converted to uncharged acetoin which can be re-consumed during the late stage of the bacterial growth [29]. Apart from this energy storage role, acetoin catabolism is proposed to play a role in the cellular NAD/NADH ratio, as its conversion is coupled to NADH formation.

### Changes in cellular energy transduction processes

The energy production and conversion category of genes (group C) was the main functional group containing the highest number of up-regulated genes during the derepression phase (Figure 3), including the hydrogenase genes previously discussed. The genes involved in cytochrome *c* biogenesis, *ccmA* (cytochrome C biogenesis protein CcmA), *ccmC* (ABC heme export system), *ccmE* (cytochrome C biogenesis protein CcmE), *ccmF* (cytochrome C biogenesis protein) and *ccmG* (cytochrome C biogenesis protein) were included in the top 50 overexpressed gene list. Cytochrome of c-type serves primarily as an electron carrier between membrane-associated components in the electron transport chain, and its maturation is a complex process driven by the genes in the *nap-ccm* operon [30]. The gene upstream of the *ccm* genes, *napA* (nitrate reductase catalytic subunit) was also on the top 50 DE gene list, and it is also included in the same *nap-ccm* operon.

Under aerobic growth, many bacteria use different types of terminal oxidases to reduce dioxygen in their respiratory pathways. The *C. necator* H16 genome encodes eight distinct terminal oxidases. Of these, the genes for five terminal oxidases were found to be up-regulated in our transcriptome analysis: *bo*_*3*_-type quinol oxidase (*cyoA2*, *cyoB2* and *cyoC2* genes), *bo*_*3*_-type chinol oxidase (*cyoA3*, *cyoB3*, *cyoC3* and *cyoD3* genes), *bb*_*3*_-type cytochrome oxidase (*coxM*, *coxN* and *coxP* genes), *bd*-type quinol oxidase subunit I (*cydA2B2*) and II (*cydB1A1*). However, the genes for *aa*_*3*_-type cytochrome oxidase (*ctaA-D* genes), *bo*_*3*_-type quinol oxidase (*cyoA1-D1* genes) and *cbb*_*3*_-type cytochrome oxidase (*ccoNPO*) were underexpressed in the derepressed cells. Induction of specific oxidases depends on the growth stage and it varies among different bacteria. For example, in *Bacillus subtilis*, it was shown that the proton-pumping cytochrome *aa*_3_ is essential for aerobic growth, as it contributes to proton motive force generation in exponentially growing cells [31].

## Conclusions

A number of proteomic and transcriptomic studies have been performed on *C. necator*, including comparing nitrogen excess/depletion for PHB expression [32], comparing lithoautotrophic growth with heterotrophic growth on succinate [21] and batch culture studies employing 3 different carbon and energy sources [22]. This unique study provides comprehensive transcriptomic data during two phases of diauxic batch growth on fructose and glycerol respectively. The importance of this study lies in the fact that this system allows for high levels of soluble hydrogenase expression under simple heterotrophic growth conditions.

Molecular hydrogen is known to be an inducer of hydrogenase genes in lithoautotrophically grown cells. Derepression of soluble hydrogenase synthesis during the metabolic switch to glycerol as the less-favoured carbon and energy source, occurs concurrently with the up-regulation of energy production and conversion systems and adaptive/stress responses associated with carbohydrate metabolic pathways. We conclude that regulation of the hydrogenase system in *C. necator* H16 is under transcriptional control, and the cellular energy deficit is the molecular signal that drives hydrogenase biogenesis under heterotrophic conditions.

## Methods

### Bacterial strain and cultivation conditions

*C. necator* H16 (DSM428, ATCC 17699) was grown heterotrophically in minimal medium (FGN) as described previously [9] with the omission of the SL-6 trace elements solution [9]. For obtaining the highest SH activity, FeCl_3_ and NiCl_2_ were added at 15 hours post-inoculation to a final concentration of 10 μM and 1 μM, respectively.

Batch fermentations were undertaken in a controlled laboratory scale glass bioreactor (Applikon, The Netherlands) with a maximum working volume of 5 litres. Growth was monitored by optical density measurement of the fermentation broth at 600 nm using a Biowave Cell Density Meter (Biochrom, England). The bioreactor was operated at 30°C with an agitation speed of 300–350 rpm and an air flow rate of 1 – 2 L/min. The initial pH of the culture was 7.0 – 7.1. Control of pH was facilitated by the automatic addition of 1 M NaOH when the pH reached the minimum set point of 6.4. Dissolved oxygen was maintained above 30% of saturation by adjusting stirrer speed.

### RNA Sample preparation and qRT-PCR

For analysing the expression of the SH genes using qRT-PCR, *C. necator* cells were harvested 11 and 34 hours after inoculation (OD_600_ of 0.6 and 6.2, respectively) from three 5 L controlled bioreactor fermentations. The cells harvested at 11 hours were deemed “pre-induction” cells, whereas the cells harvested at 34 hours were deemed “post-induction” cells. The cells were pelleted by centrifugation at 10,000 x g at 4°C for 5 min and resuspended immediately in RNA*later* (Life Technologies, USA) and stored at −80°C. Three biological replicates (three bioreactor fermentations) as well as three technical replicates were analysed for the gene expression study.

The TRIzol Plus RNA Purification Kit (Life Technologies, USA) was used to isolate total RNA. cDNA was synthesized from total RNA using the SuperScript III First-Strand Synthesis System for RT-PCR kit (Life Technologies, USA) according to the manufacturer’s protocol. Expression levels of the *hoxF*, *hypF2* and *hoxA* genes in pre- and post-induction phases were analysed using qRT-PCR to study transcriptional regulation of the SH from *C. necator*. The expression of the *gyrB* gene was used as an internal reference gene for these assays. qRT-PCR was performed on a Rotor-Gene RG-3000A cycler (Qiagen, Australia) using the SensiFAST SYBR No-ROX Kit (Bioline, Australia). A two-step cycling reaction was performed as follows: polymerase activation at 95°C for 2 min, and 40 cycles of denaturation at 95°C for 5 sec, followed by annealing/extension at 60°C for 30 sec. Data were acquired at 60°C at the completion of each extension step. The reaction was completed by melting curve analysis of a final ramp from 60°C to 95°C for qualitative monitoring of a single product. Results were analysed using the relative quantitation method (2^-ΔΔC^_T_ method) [33]. REST 2009 (Relative Expression Software Tool), standalone software based on the “Pfaffl analysis method” to estimate up and down regulation for gene expression studies was used [34] (Table 2).

**Table 2:** **Oligonucleotides used in this study**

**Primer**	**Sequence**	**Notes**
*hoxF_fwd*	CTGTTCGACACCCCCTGTAT	HoxF (NAD-reducing hydrogenase diaphorase moiety large subunit)
*hoxF_rev*	ATAGGCGATGTCCTGACTGG	
*hypF2_fwd*	CAACACCCTGGATCTGCTG	HypF2
*hypF2_rev*	GAGGATGTGGTTGAGGAAGC	
*hoxA_fwd*	CCGATTCGGAAGACATCATT	HoxA (a transcriptional activator (NtrC family) of hydrogenase genes)
*hoxA_rev*	AACTCGAGATCAAGGCGATG	
*gyrB_fwd*	GCCTGCACCACCTTGTCTTC	DNA gyrase subunit B
*gyrB_rev*	TGTGGATGGTGACCTGGATCT	

### Analysis of protein concentration and soluble hydrogenase activity

Soluble hydrogenase activity was measured according to a previously described method [10,35]. Briefly, frozen cell pellets were lysed by ultrasonication in 50 mM potassium phosphate (KPi) buffer (pH 7.0). A cuvette sealed with a Teflon septum was filled with 2.9 mL of 50 mM H_2_-saturated Tris/HCl buffer (pH 8.0), followed by addition of NAD+ (1 mM final concentration) and allowed to equilibrate to the reaction temperature (30°C). Finally, 100 μL of sample was added and NADH formation (ε = 6.22 mM^−1^ cm^−1^) was monitored at 340 nm on a Cary 100 UV-Visible Spectrophotometer with Temperature Controller (Varian, Australia). Clarified lysates were assayed for protein concentration using the Direct Detect™ infrared-based spectrometer (Millipore, USA).

### Sample preparation for Illumina sequencing

DNase-treated total RNA was depleted of rRNA with the Ribo-Zero magnetic kit (Epicenter, USA). The quality of the total RNA was evaluated using the Agilent 2100 Bioanalyzer (Agilent Technologies, USA). mRNA-Seq was performed by the Ramaciotti Centre for Genomics, UNSW. For the mRNA-Seq sample preparation, the Illumina standard kit was used, according to the manufacturer’s protocol (Illumina, USA).

### Transcriptome sequencing and RNA-Seq analysis

After 11 hours cultivation, the cells were assumed to be in the exponential growth phase where the hydrogenase genes are repressed, whereas after 34 hours the hydrogenase genes are derepressed when glycerol is a main carbon source. Two technical replicates of each growth phases from two independent biological experiments were withdrawn for transcriptome analyses. The eight samples were sequenced using the llumina HiSeq 2000 following the standard Illumina protocol using a 36-bp read length, in the Ramaciotti Centre for Genomics, the University of New South Wales.

Prior to RNA-Seq analysis, filters were applied to remove low quality reads from all eight pair-end samples. Pair-end raw reads were trimmed with the BWA trimming mode at a threshold of Q13 (P = 0.05) as implemented by SolexaQA version 1.11 [36]. Low-quality 3′ ends of each read were filtered. Reads that were less than 25 bp in length were discarded.

Filtered reads from eight samples were aligned to the genomic sequence of *C. necator* H16 (NCBI accession number NC_008313.1, NC_008314.1 and NC_005241.1) with TopHat 2.0.4 [37] and Bowtie 2–2.0.0-beta7 [38] using default parameters. At least 98.9% of reads from each sample were mapped to the reference genome. Count files of the aligned sequencing reads were generated by the htseq-count script from the Python package HTSeq [39] with intersection-nonempty mode, using the GFF annotation file downloaded from NCBI. The read counts from each sequenced sample were combined into a count file, which was subsequently used for the differential expression analysis. In the count table, each row represents a gene from the NCBI genome reference, each column a sequence RNA library, and the values give the raw numbers of sequencing reads that were mapped to the respective gene in each library. Differential analysis was performed to the count files using edgeR [40] and DESeq2 [41] packages, following standard normalization procedures. Respectively by both packages, the count data was rescaled. Genes with less than 10 total counts in both conditions were removed from further analysis.

Only genes differentially expressed at a FDR < = 0.1 and a P value <0.05 were considered as significantly differentially expressed (SDE) genes in both analysis. DE genes reported by both edgeR and DESeq2 were considered for further analysis. The lists of up and down regulated SDE genes were placed into COG categories respectively by NCBI conserved domain search [42].

## Additional files

Additional file 1: Table S1. The up-regulated genes during the hydrogenase de-repression condition (growth on glycerol) via RNA-seq analysis of *Cupriavidus necator* H16. Additional file 2: Table S2. The down-regulated genes during the hydrogenase de-repression condition (growth on glycerol via RNA-seq analysis of *Cupriavidus necator* H16.
